# Europe PMC in 2020

**DOI:** 10.1093/nar/gkaa994

**Published:** 2020-11-12

**Authors:** Christine Ferguson, Dayane Araújo, Lynne Faulk, Yuci Gou, Audrey Hamelers, Zhan Huang, Michele Ide-Smith, Maria Levchenko, Nikos Marinos, Rakesh Nambiar, Maaly Nassar, Michael Parkin, Xingjun Pi, Faisal Rahman, Frances Rogers, Yogmatee Roochun, Shyamasree Saha, Mohamed Selim, Zunaira Shafique, Shrey Sharma, David Stephenson, Francesco Talo', Arthur Thouvenin, Santosh Tirunagari, Vid Vartak, Aravind Venkatesan, Xiao Yang, Johanna McEntyre

**Affiliations:** Literature Services, EMBL-EBI, Wellcome Trust Genome Campus, Cambridge, UK; Literature Services, EMBL-EBI, Wellcome Trust Genome Campus, Cambridge, UK; Literature Services, EMBL-EBI, Wellcome Trust Genome Campus, Cambridge, UK; Literature Services, EMBL-EBI, Wellcome Trust Genome Campus, Cambridge, UK; Literature Services, EMBL-EBI, Wellcome Trust Genome Campus, Cambridge, UK; Literature Services, EMBL-EBI, Wellcome Trust Genome Campus, Cambridge, UK; Literature Services, EMBL-EBI, Wellcome Trust Genome Campus, Cambridge, UK; Literature Services, EMBL-EBI, Wellcome Trust Genome Campus, Cambridge, UK; Literature Services, EMBL-EBI, Wellcome Trust Genome Campus, Cambridge, UK; Literature Services, EMBL-EBI, Wellcome Trust Genome Campus, Cambridge, UK; Literature Services, EMBL-EBI, Wellcome Trust Genome Campus, Cambridge, UK; Literature Services, EMBL-EBI, Wellcome Trust Genome Campus, Cambridge, UK; Literature Services, EMBL-EBI, Wellcome Trust Genome Campus, Cambridge, UK; Literature Services, EMBL-EBI, Wellcome Trust Genome Campus, Cambridge, UK; Literature Services, EMBL-EBI, Wellcome Trust Genome Campus, Cambridge, UK; Literature Services, EMBL-EBI, Wellcome Trust Genome Campus, Cambridge, UK; Literature Services, EMBL-EBI, Wellcome Trust Genome Campus, Cambridge, UK; Literature Services, EMBL-EBI, Wellcome Trust Genome Campus, Cambridge, UK; Literature Services, EMBL-EBI, Wellcome Trust Genome Campus, Cambridge, UK; Literature Services, EMBL-EBI, Wellcome Trust Genome Campus, Cambridge, UK; Literature Services, EMBL-EBI, Wellcome Trust Genome Campus, Cambridge, UK; Literature Services, EMBL-EBI, Wellcome Trust Genome Campus, Cambridge, UK; Literature Services, EMBL-EBI, Wellcome Trust Genome Campus, Cambridge, UK; Literature Services, EMBL-EBI, Wellcome Trust Genome Campus, Cambridge, UK; Literature Services, EMBL-EBI, Wellcome Trust Genome Campus, Cambridge, UK; Literature Services, EMBL-EBI, Wellcome Trust Genome Campus, Cambridge, UK; Literature Services, EMBL-EBI, Wellcome Trust Genome Campus, Cambridge, UK; Literature Services, EMBL-EBI, Wellcome Trust Genome Campus, Cambridge, UK

## Abstract

Europe PMC (https://europepmc.org) is a database of research articles, including peer reviewed full text articles and abstracts, and preprints - all freely available for use via website, APIs and bulk download. This article outlines new developments since 2017 where work has focussed on three key areas: (i) Europe PMC has added to its core content to include life science preprint abstracts and a special collection of full text of COVID-19-related preprints. Europe PMC is unique as an aggregator of biomedical preprints alongside peer-reviewed articles, with over 180 000 preprints available to search. (ii) Europe PMC has significantly expanded its links to content related to the publications, such as links to Unpaywall, providing wider access to full text, preprint peer-review platforms, all major curated data resources in the life sciences, and experimental protocols. The redesigned Europe PMC website features the PubMed abstract and corresponding PMC full text merged into one article page; there is more evident and user-friendly navigation within articles and to related content, plus a figure browse feature. (iii) The expanded annotations platform offers ∼1.3 billion text mined biological terms and concepts sourced from 10 providers and over 40 global data resources.

## INTRODUCTION

Europe PMC is an open repository of research publications, supporting the Open Access policies of 31 international funders of life sciences research. The repository is built in collaboration with the PMC archive in the USA and contains over 6 million full text articles and 37 million abstracts. Incoming full text articles are shared between the two sites daily. Since 2018, Europe PMC has also indexed preprint abstracts and recently started including the full text of Covid-19-related preprints.

In addition to providing access to the core publications, Europe PMC adds value in a number of ways. For example, Europe PMC is a major integrator of ORCIDs (https://orcid.org/), data, open citations, text-mined concepts and data citations, and grant information.

The content in Europe PMC is updated daily and can be searched and retrieved via the website (https://europepmc.org) and API (Application Programming Interface; https://europepmc.org/RestfulWebService). Content is also available in bulk via FTP downloads (https://europepmc.org/downloads). Europe PMC is part of the global life science data infrastructure. In addition to sharing content with the USA National Library of Medicine, which runs PubMed and PMC, Europe PMC is an ELIXIR Core Data Resource (https://www.elixir-europe.org/platforms/data/core-data-resources) (1) and provides integration with over 60 critical data resources, such as the Protein Data Bank in Europe (2), the European Nucleotide Archive (3) and UniProt (4).

The most significant changes to Europe PMC since our previous report (5) will be elaborated in this article. These involve the addition of preprints, a website redesign and extension of the text mining publishing and display platform, SciLite (6). The first significant change we report is the addition of life science preprint abstracts to Europe PMC’s core content, making it possible to search across 15 preprint servers for pre-peer review versions of biomedically relevant research papers. Secondly, infrastructure changes that improve the usability and performance of the website and search engine, were brought about by a website redesign in 2019. Notably, this resulted in displaying records with merged abstracts & full text pages and made it easier to find related information through changes to the ‘search’ and ‘article’ pages. The third significant change has been to build on the text mining platform and expand the results that can be displayed through SciLite: text mining and machine learning approaches hold promise to improve the discoverability of the research literature. Europe PMC continues to support the text mining community in this endeavour, now offering a submission system for text mined results and by providing a platform for the reuse of text-mined annotations on open access content. Furthermore, links continue to be built to additional data and new resources behind the articles.

Finally, given the coronavirus pandemic of 2020, Europe PMC has worked to make several key COVID-19 resources available. In addition to ingesting the full text of COVID-19 -related preprints, Europe PMC’s journal-published content includes additional COVID-19 articles released from embargo and made available by all major publishers participating in the Public Health Emergency COVID-19 Initiative. See this blog for more information: https://www.nih.gov/news-events/news-releases/national-library-medicine-expands-access-coronavirus-literature-through-pubmed-central Beyond this, Europe PMC is ingesting and provides search functionality for COVID-19-related awarded grants from 25+ funding agencies. Europe PMC also contributes literature links to The European COVID-19 Data portal (Cantelli G et al. (2020) The European Bioinformatics Institute: Empowering Cooperation in response to a Global Health Crisis. Submitted).

### Preprints

Europe PMC has been indexing preprint abstracts since 2018—over 180 thousand preprint abstracts currently sit among all of journal published articles hosted in the database, including >31 million abstracts (mostly from PubMed) and 6 million full text articles (https://europepmc.org/About). This is significant because researchers can now search in one place to find biomedical preprints aggregated from leading preprint servers, improving their discoverability.

Europe PMC’s criteria for indexing content from a preprint server includes the following: the server offers content relevant to biomedical research, has a publicly available screening process for preprints, and for each preprint offers information typical of a research article, ideally: server name, article title, author names and affiliations, abstract, posting date, article identifier. In addition, versioning should be supported, and the version information should be available in metadata, so that versions can be supported in Europe PMC. Preprints should be clearly identifiable on the servers as having not been peer reviewed, and/or clearly identify review status, both on website displays and in machine readable form.

**Figure 1.**
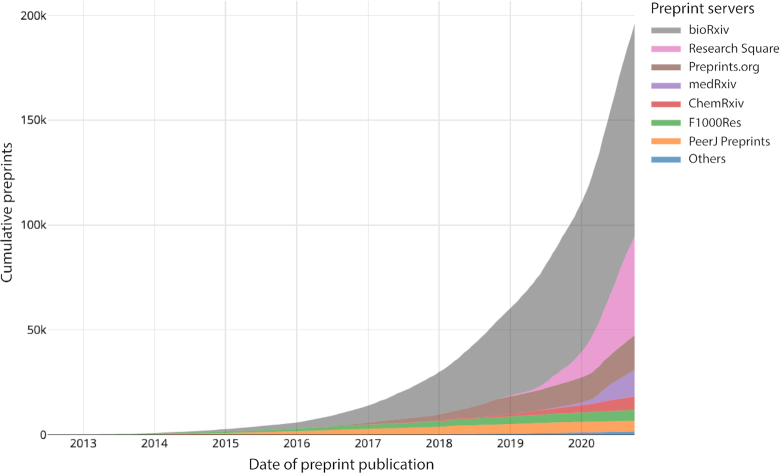
Preprints in Europe PMC (Accessed September 2020).

Europe PMC currently indexes preprints from 15 life science servers including BioRxiv (https://www.biorxiv.org/), MedRxiv (https://www.medrxiv.org/), Research Square (https://www.researchsquare.com/), F1000 Research (https://f1000research.com/) and seven F1000-powered Open Research platforms, including Wellcome Open Research (https://wellcomeopenresearch.org/) and Gates Open Research (https://gatesopenresearch.org/) (Figure 1). The team behind Europe PMC are working to include preprints from other servers.

**Figure 2.**
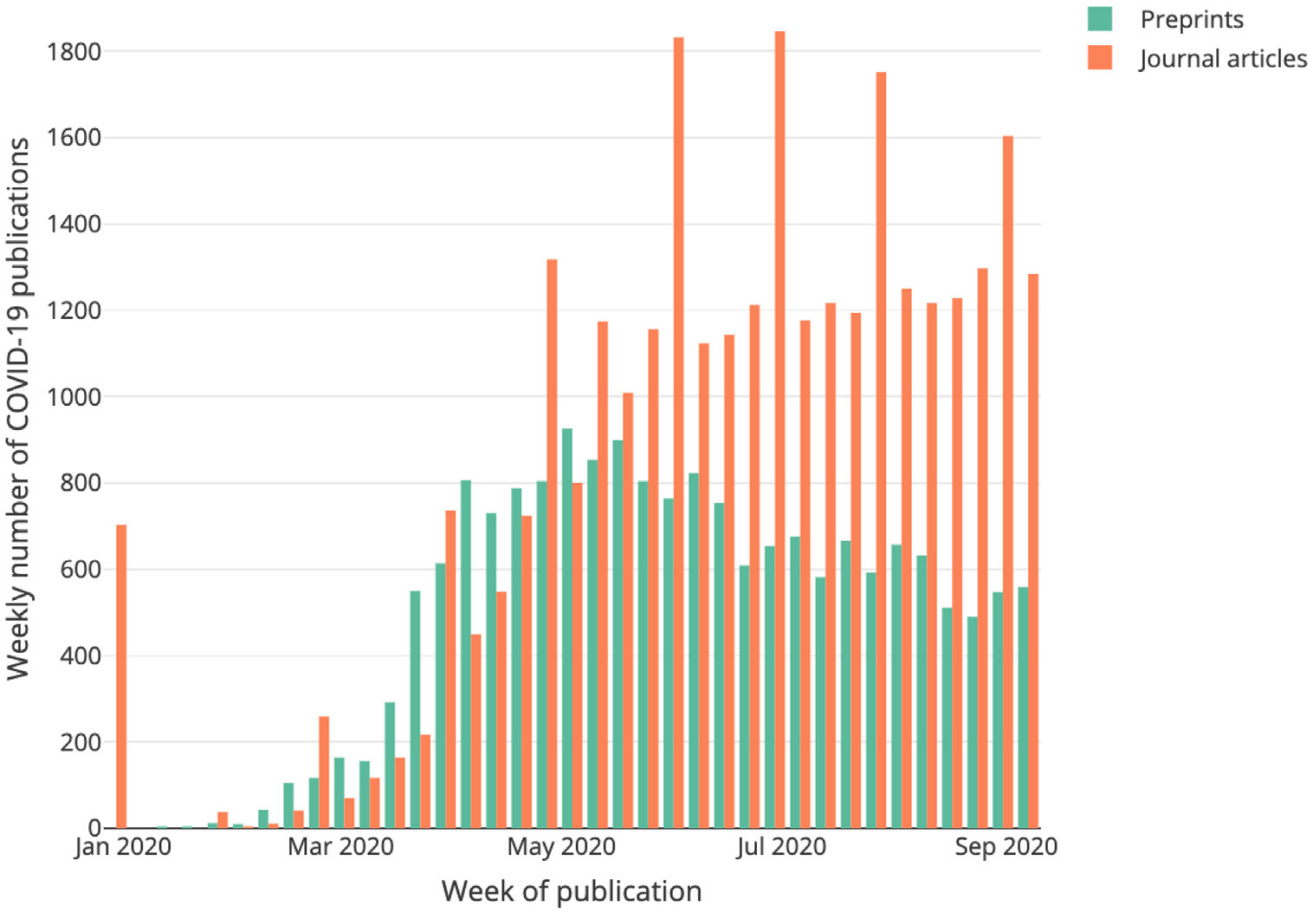
COVID-19 preprints indexed per week (in green) vs peer-reviewed journal articles (in orange) in Europe PMC (accessed September 2020)

The need to improve preprint distribution has been brought sharply into focus by the coronavirus pandemic. About one-third of COVID-19 research is still available only in preprints (Figure 2).

In 2020, the collection of preprint abstracts indexed in Europe PMC was extended to include the full text of COVID-19-related preprints, making them openly available alongside the full text of COVID-19 journal articles in Europe PMC, improving their discoverability and potentially accelerating scientific understanding of COVID-19.

Being typically scattered across a number of platforms and only available in PDF format means that this COVID-19 preprinted research is significantly harder to find, read, reuse and text-mine than journal articles. Supported by Wellcome in partnership with the UK Medical Research Council (MRC) and the Swiss National Science Foundation (SNSF), Europe PMC is now ingesting the full text of COVID-19 preprints and making them available to researchers and machines for reading/text mining and reuse in a standard XML format, alongside peer reviewed full text articles. This initiative will accelerate scientific research on COVID-19, provide opportunities to build on open and rapid publication systems, and form a corpus for future history-of-science research.

**Figure 3.**
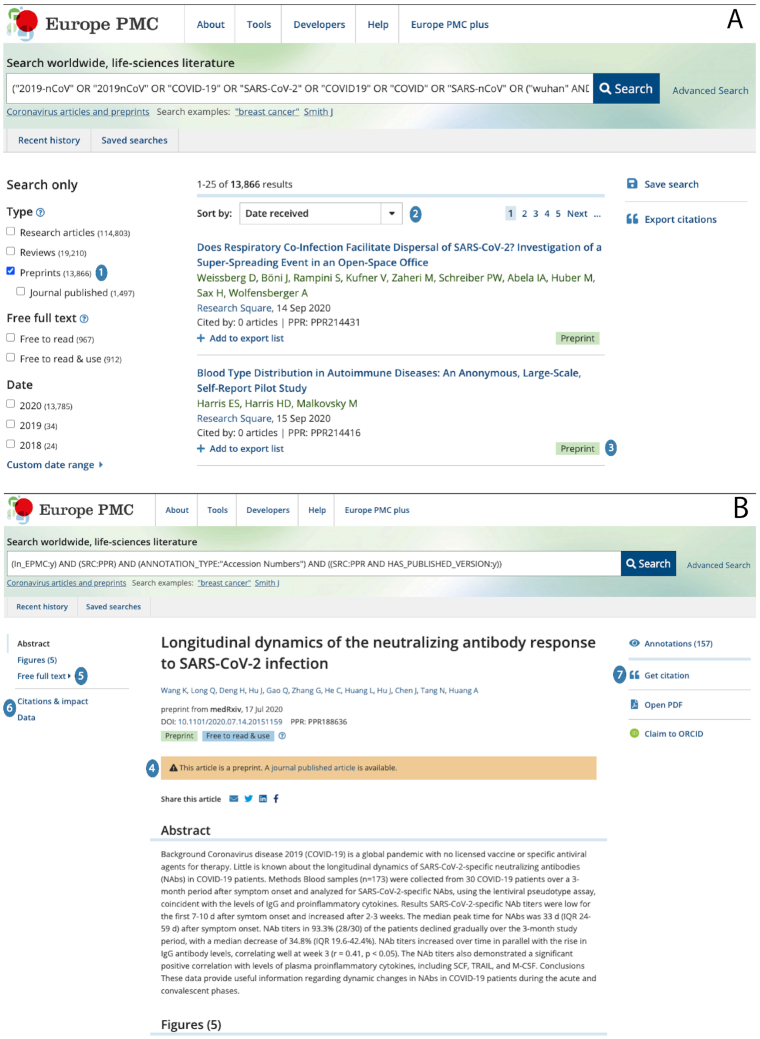
Display of preprints in Europe PMC’s redesigned search page (A) and article page (B). Key for 1–7 can be found in the main text. A bespoke COVID-19 search link can be found under the search box. This automatically populates the search box with the Boolean terms required to identify all coronavirus-related content in Europe PMC. Search functionality includes filters for content type, free full text and date (left), the list of search results is in the main view; actions, such as saving a search or exporting citations, are displayed to the right. Web pages accessed September 2020.

Given that preprints are technically the same as journal-published articles, Europe PMC re-uses the same infrastructure to ingest, index, display and enrich them. See Figure 3 for the display of preprint content in Europe PMC’s recently redesigned web pages. The redesigned search page (Figure 3A) makes it possible to filter searches for research articles, reviews or preprints [label 1]. Preprints can be filtered further for those linked to journal published versions. The results returned by a search query in the main view can be sorted by date received, date published, times cited etc [label 2]. Preprint content in the search results is clearly marked with a green ‘preprint’ tag [label 3].

Preprints are differentiated from peer-reviewed articles in Europe PMC. As seen in the article page for a typical preprint (Figure 3B), where a subsequent journal-published version of a preprint exists, the preprint and journal article are linked [label 4]. The full text of the preprint can be accessed by links in the navigation panel on the left [label 5]. The preprint has been enriched with cross links as for all journal articles. In this case links to data behind the article, citation and alternative metrics are available via the navigation panel on the left [label 6]. To date, more than 9,9 thousand preprints in Europe PMC have been cited at least once. In the navigation panel to the right of the web page [label 7], a panel of 157 annotations (text mined terms) is available to view, as is the ability for an author to add this preprint to their ORCID profile.

### The new Europe PMC website

A major website redesign in 2019 merged the abstracts and full text pages of the indexed articles making it easier to find related information via the ‘search’ page and ‘article’ page (Figure 3).

Logistically, information for the abstract and full text article comes into Europe PMC from different sources (e.g. abstracts are ingested from PubMed or preprint servers whereas full text articles are ingested from PMC, or Europe PMC’s own submission system). Full text articles are richer than abstracts (for instance, text mining a full text article for biological concepts such as gene-disease relationships would return many more results than the abstract). Merging the information that Europe PMC holds for the abstract and the full text into one location, means that both will be discovered by a single search and even if licensing won’t permit display of the full text, the text mining results will be searchable and the article discovered through the abstract.

#### The enriched article

**Figure 4.**
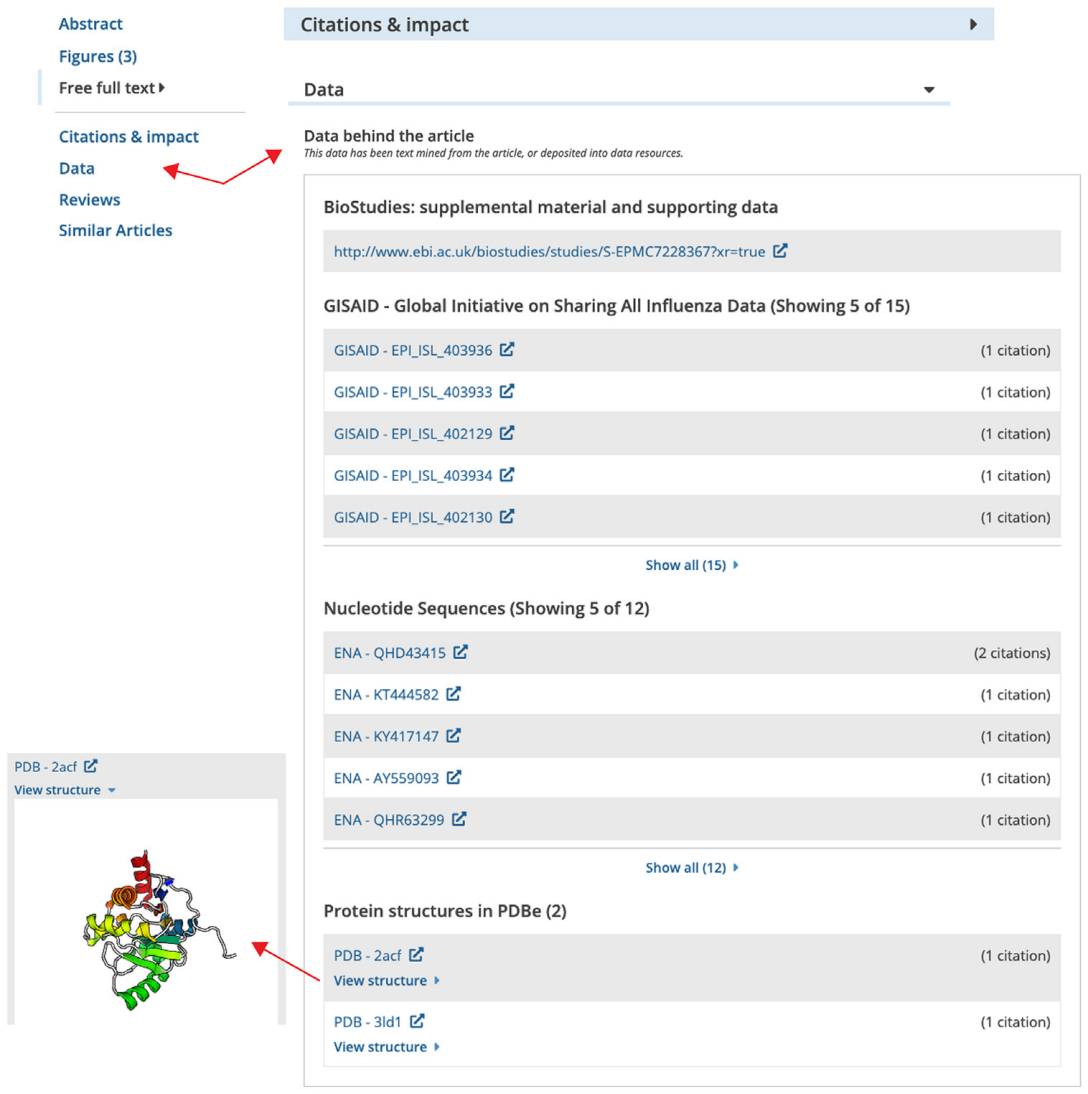
The data tab in the redesigned article page. The left hand navigation panel leads to the categories of research resources available for https://europepmc.org/article/MED/32083328#data (accessed September 2020).

All of the content related to the article is provided on the same page, including the abstract, full text, data, and other useful resources. Europe PMC has put special emphasis on access to data, making it easier to get an overview of the key findings. This is now supported by two dedicated sections: ‘figures’ and ‘data’, that can be reached using the left-hand navigation menu on the article page (Figure 4).

Figures for the open access articles are now available to preview directly under the abstract. You can view them as thumbnails, click to get an expanded view including the figure legend, or use the full text link to view a figure of interest in context.

**Figure 5.**
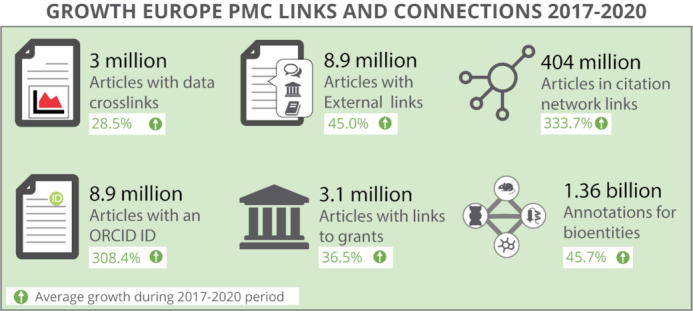
The growth of crosslinked research resources offered by Europe PMC 2017–2020: Europe PMC’s search engine is more powerful because of the links between journal articles/preprints and related research resources.

The ‘data’ section is the go-to place for all research data associated with a study. It contains links to supplemental, supporting, and related or curated data. ‘Data behind the article’ combines the mentions in the text of an article of data accessions and DOIs, for over 60 different life science databases (Supplementary Table S1). The reciprocal, ‘data links’ are citations of an article by a database record. All data mentions are linked to external resources, where you can find more information about the data itself. Over 3 million publications now link to related data, an increase of 28.5% since 2017 (see Figure 5). The number of referrals to Europe PMC from EMBL-EBI data resources has also increased by several orders of magnitude from 12 thousand per month to 300 thousand per month over the past 4 years.

The list of full text article sections available for deep searching has been extended to include a ‘Data Availability’ category. This captures the different name variants used for sections in an article that acknowledge where data is made available. Articles containing a data availability statement can be found using ‘DATA_AVAILABILITY:*’ syntax as the search query. See this blog for more information: http://blog.europepmc.org/2018/11/mapping-out-path-to-data.html.

Europe PMC has collaborated with 55 different providers to link preprints and peer reviewed publications to useful resources that provide free access to peer reviews, recommendations, protocols and materials, lay summaries and more. Currently 8.9 million articles in Europe PMC feature these ‘external links’, a number that has increased by 45% since 2017 (see Figure 5).

The ‘reviews’ section houses post-publication peer reviews and scientific commentaries from services such as Faculty opinions (formerly, F1000Prime; https://facultyopinions.com/prime/home), preLights (https://prelights.biologists.com/about-us/), Peer Community In (https://peercommunityin.org/) and Publons (https://publons.com/); while the ‘Protocols and Materials’ section includes additional information about cell lines, reagents or methods used in the article.

The Impact section (in the ‘Citations & Impact’ tab) contains not only traditional article citations and alternative metrics, but where present, shows data citations and article recommendations by experts. Article citations in Europe PMC are those from both the full text articles in its collection and the open citation network where the reference lists of articles are made openly available by publishers *and* where the citing articles have unique identifiers such as PMC identifiers. There are currently 404 million articles in the citation network, having grown by 333% since 2017—in large part due to the Initiative for Open Citations (https://i4oc.org). See Figure 5.

**Figure 6.**
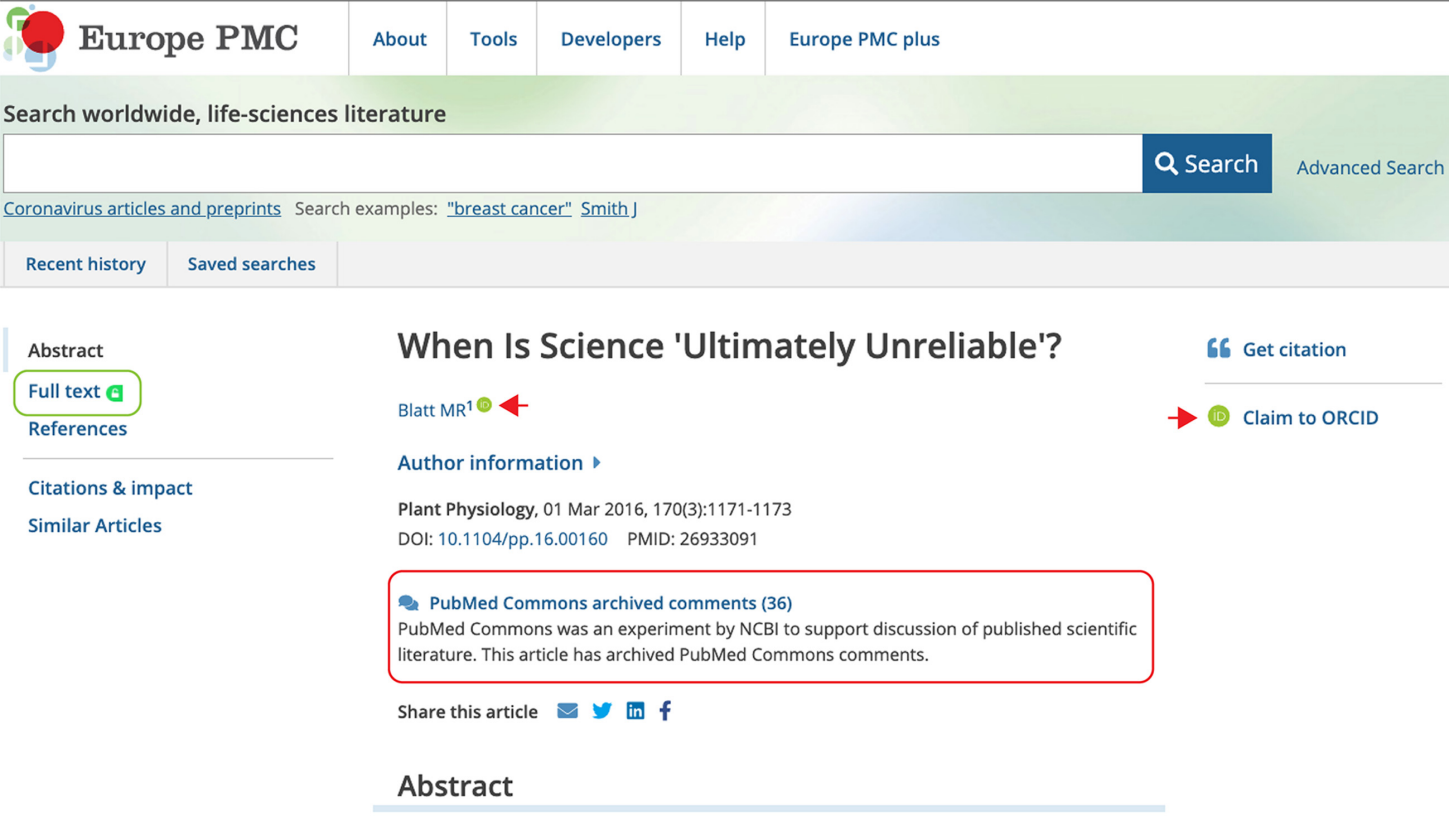
The article page in Europe PMC: Integrations with Unpaywall (green lozenge) provide links to a free legal copy of the full text. Links to PubMed Commons comments, where available, are displayed above the abstract (red lozenge). ORCID integration is possible via the ‘claim to ORCID’ feature (red arrow, right); once claimed, the ORCID icon appears alongside the author's name (red arrow, left). Web page accessed September 2020.

These value-add research resources can be found in a user-friendly section on the article page categorised into supporting data, along with reviews, metrics, protocols and funding acknowledgements, where present. Additional features of the article page are captured in Figure 6.

#### The grant finder tool and grants database

Europe PMC maintains a grants database (see https://europepmc.org/grantfinder) that holds funding details for grants awarded by its 31 European research funders (https://europepmc.org/Funders/) so that articles and grants can be linked (Figure 5). Grant-publication associations are supplied directly by funders, via Researchfish (https://researchfish.com/), or by the fundees, when using the Europe PMC manuscript submission system (https://plus.europepmc.org/).

In addition, Europe PMC routinely text mines the *Acknowledgements* and *Funding* sections of incoming articles for grants awarded by Europe PMC funders. This added a further 27.6 thousand articles links to grants in 2019, after text mining for papers published between 2006 and 2018.

DOIs for grants: in 2019, Europe PMC began to incorporate DOIs for Wellcome grants held in the database. To participate in this unique global identifier system for grants, funders register DOIs with Crossref, where they deposit their grant metadata (https://www.crossref.org/community/funders/ - registering-research-grants). The funders also need to ensure there are openly available web pages with grant information for readers, to which the grant DOIs link.

**Figure 7.**
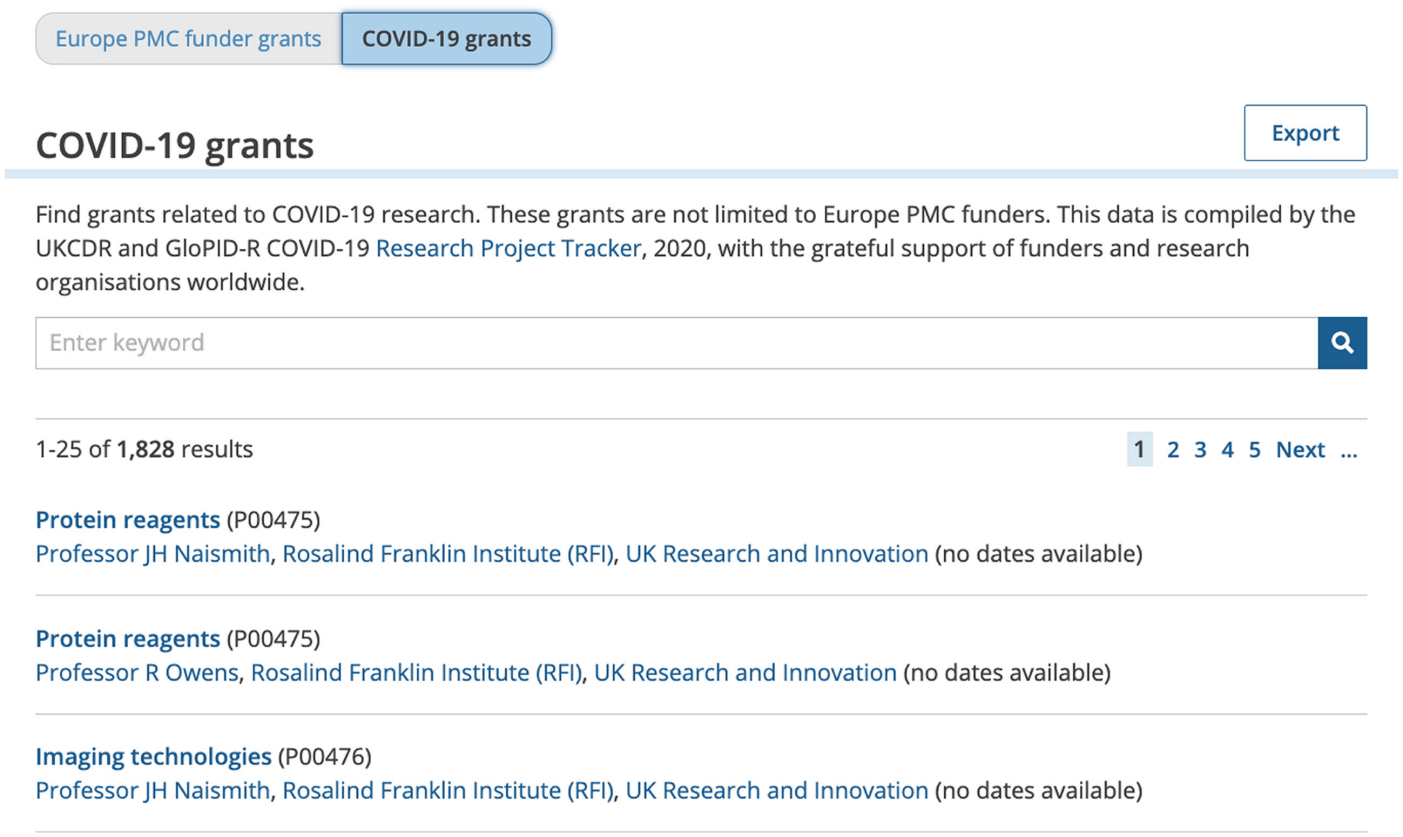
The user interface of grant finder tool. The tool can be used to search for details of more than 80 thousand grants from Europe PMC funders or more than 18 hundred COVID-19 grants. https://europepmc.org/grantfinder (accessed September 2020.)

Tracking COVID-19 research grants: Details of COVID-19 research awards from any funder are now included in the Europe PMC grants database (Figure 7). Europe PMC worked with the MRC and the UKCDR and GloPID-R dataset to include these grants, which can be linked to preprints and journal articles as they become available in Europe PMC. When the first set of grants were uploaded in July 2020, they numbered 1800+, and totalled >$700 million from 25+ funding agencies. See this blog for more information: https://mrc.ukri.org/news/blog/mapping-the-covid-19-research-landscape-how-tracking-medical-research-funding-has-accelerated-in-the-pandemic/.

### Text mining to expose research terms and concepts

#### The growing text mining platform

**Figure 8.**
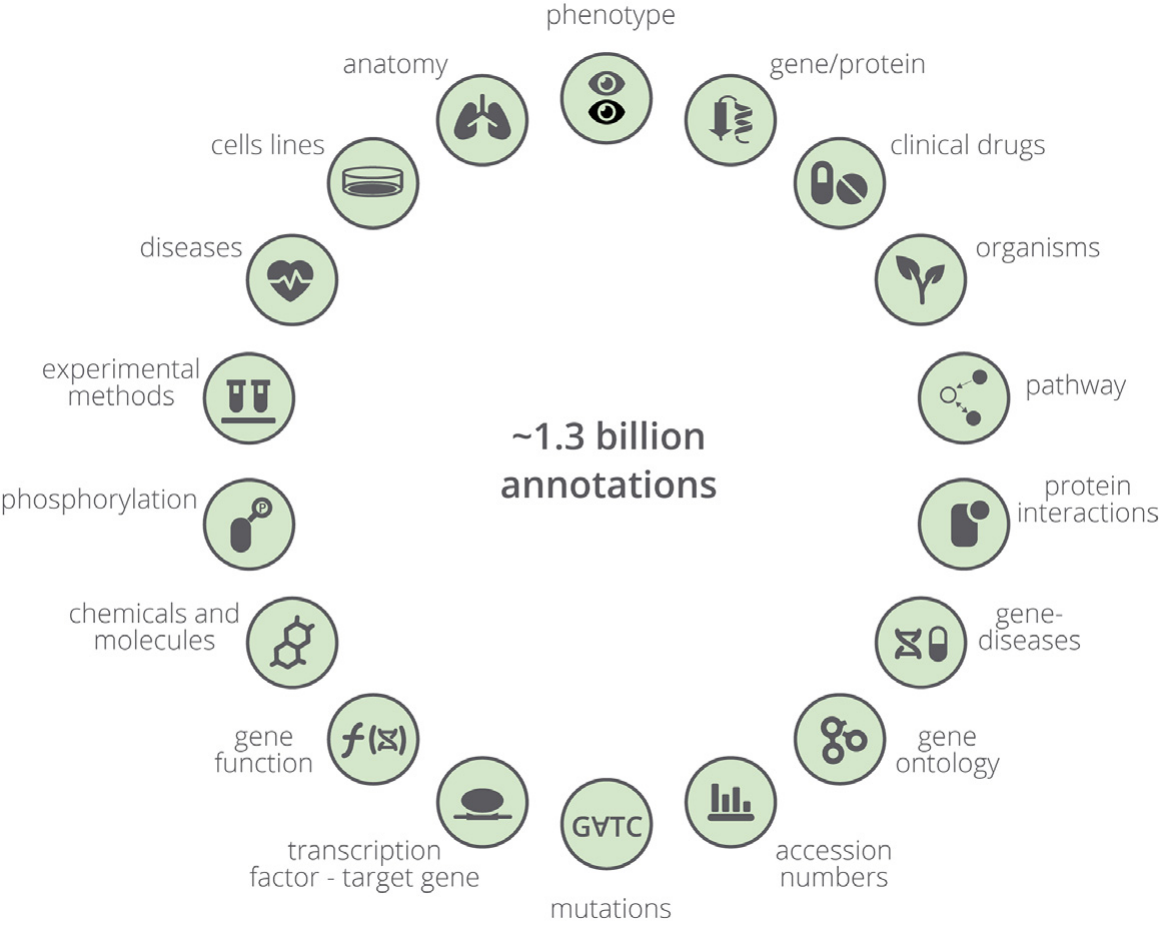
Annotation types: the biological terms and concepts that are currently text mined from articles indexed in Europe PMC.

The Europe PMC Annotations platform is a community platform established to capitalise on the advances made by the text-mining community, bringing the results to a broader scientific community. The open architecture of the platform, based on standard data format (Web Annotation Data Model https://www.w3.org/TR/annotation-model/), allows text-mining outputs from different sources to be integrated and reused. The platform is flexible with regard to the frequency of annotation deposition from providers: anything from daily updates to a one-off bulk deposition can be accommodated. Annotations can be uploaded automatically via a submission system, a new feature added to the platform in 2019. The platform currently contains ∼1.3 billion annotations sourced from 10 providers and has been expanded to cover bio entities ranging from, for example, accession numbers (data citations) from over 60 global data resources (Supplementary Table S1), curated text snippets on protein-protein interactions from IntAct database (7), to Open Targets gene-disease relationships (8) (Figure 8). Furthermore, a mechanism that enables deep links between curated databases and research publications is now available. The Annotations API allows users to programmatically access the annotations hosted by the Annotations platform (http://www.europepmc.org/AnnotationsApi). The API offers functionalities that support queries, such as retrieving annotations associated with a specified list of articles; or retrieving annotations of a given type from a specified article section, for example all chemicals mentioned in ‘Materials and Methods’.

#### SciLite to highlight text annotations

SciLite is an application developed to help users to scan articles by highlighting key biological entities, such as genes/proteins, diseases and experimental methods in content (6). The SciLite application has been improved since 2017 by increasing the annotation accuracy and the types of annotated terms offered. Related open source code is available on GitHub (see DATA AVAILABILITY). The website functionality has also been improved: the annotations panel that is included alongside each article now offers greater granularity of the annotated terms and has been redesigned to encourage increased access and greater speed of information retrieval. More technical users will find improved data available through the annotations API (e.g. greater granularity of article sections offered for query). Moreover, files of mined accession numbers on the FTP site are now deposited weekly instead of monthly.

## CONCLUDING COMMENTS

Since 2017 Europe PMC has achieved three major new developments: ingesting preprint abstracts and the full text of COVID-19 preprints; a website redesign and extensions to the text mining publishing and display platform, SciLite. Over the next 2–3 years Europe PMC plans to continue delivering a robust archiving and discovery service and integrating the literature with data and other resources. Europe PMC will build on its work ingesting and displaying preprint versions and linking them to published, peer-reviewed articles, for example by making the availability and status of peer review materials more transparent. By January 2021 Europe PMC will fully support the Plan S Open Access policies of the Europe PMC funders who have signed up to Coalition S, to ensure that their researchers can comply with these new policies (https://www.coalition-s.org/). Europe PMC will also investigate innovative ways of reusing text-mined annotations for search and discovery.

## DATA AVAILABILITY

Europe PMC’s annotation validator and Identifier extractor is an open source collaborative initiative available in the GitHub repository (https://github.com/EuropePMC/EuropePMC-Annotation-Validator and https://github.com/EuropePMC/EuropePMC-Identifier-Extractor).

## Supplementary Material

gkaa994_Supplemental_File

## References

[1] Durinx C., McEntyre J., Appel R., Apweiler R., Barlow M., Blomberg N., Cook C., Gasteiger E., Kim J.H., Lopez R. et al. Identifying ELIXIR Core Data Resources [version 2; referees: 2 approved]. F1000Research. 2017; 5:2422. 10.12688/f1000research.9656.1 PMC5070591 27803796

[2] Mir S., Alhroub Y., Anyango S., Armstrong D.R., Berrisford J.M., Clark A.R., Conroy M.J., Dana J.M., Deshpande M., Gupta D. et al. PDBe: towards reusable data delivery infrastructure at protein data bank in Europe. Nucleic Acids Res. 2018; 46:D486–D492. 29126160 10.1093/nar/gkx1070 PMC5753225

[3] Amid C., Alako B.T.F., Burdett B.K.V.T., Burgin J., Fan J., Harrison P.W., Holt S., Hussein A., Ivanov E. et al. The European Nucleotide Archive in 2019. Nucleic Acids Res. 2020; 48:D70–D76. 31722421 10.1093/nar/gkz1063 PMC7145635

[4] UniProt Consortium UniProt: a worldwide hub of protein knowledge. Nucleic Acids Res. 2019; 47:D506–D515. 30395287 10.1093/nar/gky1049 PMC6323992

[5] Levchenko M., Gou Y., Graef F., Hamelers A., Huang Z., Ide-Smith M., Iyer A., Kilian O., Katuri J., Kim J.-.H. et al. Europe PMC in 2017. Nucleic Acids Res. 2018; 46:D1254–D1260. 29161421 10.1093/nar/gkx1005 PMC5753258

[6] Venkatesan A., Kim J.H., Talo F., Ide-Smith M., Gobeill J., Carter J., Batista-Navarro R., Ananiadou S., Ruch P., McEntyre J. SciLite: a platform for displaying text-mined annotations as a means to link research articles with biological data. Wellcome Open Re. 2016; 2:25. 10.12688/wellcomeopenres.10210.2 PMC5527546 28948232

[7] Kerrien S., Aranda B., Breuza L., Bridge A., Broackes-Carter F., Chen C., Duesbury M., Dumousseau M., Feuermann M., Hinz U. et al. The IntAct molecular interaction database in 2012. Nucleic Acids Res. 2012; 40:D841–D846. 22121220 10.1093/nar/gkr1088 PMC3245075

[8] Carvalho-Silva D., Pierleoni A., Pignatelli M., Ong C.K., Fumis L., Karamanis N., Carmona M., Faulconbridge A., Hercules A., McAuley E. et al. Open Targets Platform: new developments and updates two years on. Nucleic Acids Res. 2019; 47:D1056–D1065. 30462303 10.1093/nar/gky1133 PMC6324073

