# Supplementary material for: Europe PMC in 2020
Source: Nucleic Acids Res. 2020 Nov 12;49(D1):D1507–14. doi: 10.1093/nar/gkaa994 (PMC7778976; doi:10.1093/nar/gkaa994)
Supplement: gkaa994_Supplemental_File [file gkaa994_supplemental_file.pdf]

	Europe PMC mines accession numbers & resource names for the databases listed.	
**Resource(s)**	**Accession number**	**Resource name**
ArrayExpress	●	●
BioModels	●	●
BioProject	●	
BioSamples	●	●
BioStudies	●	●
BRENDA		●
CATH	●	●
ChEBI	●	●
ChEMBL	●	●
Complex Portal	●	●
dbGap	●	
DOI	●	
EBI Metagenomics	●	●
EBiSC	●	
EFO	●	●
EGA	●	●
EMDB	●	●
EMPIAR	●	●
ENA	●	●
Ensembl	●	●
Ensembl Genomes		●
Enzyme Portal		●
EudraCT	●	
Europe PMC		●
EVA	●	●
Expression Atlas		●
GCA	●	
GEO	●	
GISAID	●	
GO	●	●
GWAS Catalog		●
HGNC	●	●
HipSci	●	
Human Protein Atlas	●	●
Identifiers.org		●
IGSR/1000 genomes	●	●
Intact	●	●
Intenz	●	●
InterPro	●	●
Metabolights	●	●
MINT	●	●
Mouse Resources	●	●
NIH ClinicalTrials	●	
OLS		●
OMIM	●	
Orphadata	●	●
PDBe	●	●
Pfam	●	●
PRIDE/ProteomeXchange	●	●

Reactome	•	•
RefSeq	•	
RefSNP	•	
Rfam	•	•
RNACentral	•	•
RRID	•	
SILVA		•
STRING-db		•
SureChEMBL		•
Treefam	•	
UniProt	•	•
VectorBase		•
WormBase		•
